# Effectiveness and quality of life improvement in young adult schizophrenia patients treated with Abilify Maintena

**DOI:** 10.1192/j.eurpsy.2023.2228

**Published:** 2023-07-19

**Authors:** D. Petrić, A. Kašelan, S. Brozan, M. Žuljan Cvitanović, D. Krnić

**Affiliations:** 1 Department of Child and Adolescent Psychiatry, Clinical Hospital Centre Rijeka, Rijeka; 2 Department of psychiatry, University Hospital Centre, Split, Croatia

## Abstract

**Introduction:**

Treating young patients with schizophrenia is a challenge, as these patients have much to gain from controlled pharmacotherapy and even more to lose with a possible relapse. Treating patients with long-acting injectable antipsychotics avoids the issue of non-compliance, the biggest risk factor for relapse, while also improving the quality of life. Receiving a drug once-a-month can provide greater flexibility and convenience to our patients.

**Objectives:**

Aim of the paper is to assess the efficacy and quality of life in monthly dosing of long-acting injectable antipsychotic in young adult schizophrenia patients.

**Methods:**

The research included 7 patients aged 19 to 25 years who were diagnosed in accordance with the Diagnostic and Statistical Manual of Mental Disorders, Fifth Edition. Patients were assessed eight times over two years using the following clinical scales: Positive and Negative Syndrome Scale, Clinical Global Impression – Severity and Improvement Scale, Treatment Satisfaction Questionnaire for Medication (TSQM-9) and Quality of Life Scale (QOLS).

**Results:**

All treated patients achieved remission. There was a statistically significant improvement in measured scales in all patients. There were no side-effects reported during the study period, with no relapse or new hospitalizations

**Conclusions:**

The monthly formulation of aripiprazole has proven to be effective and safe in our study and has great potential to improve patient quality of life as well.

**Disclosure of Interest:**

None Declared

